# Mammographically dense human breast tissue stimulates MCF10DCIS.com progression to invasive lesions and metastasis

**DOI:** 10.1186/s13058-016-0767-4

**Published:** 2016-10-25

**Authors:** Cecilia W. Huo, Mark Waltham, Christine Khoo, Stephen B. Fox, Prue Hill, Shou Chen, Grace L. Chew, John T. Price, Chau H. Nguyen, Elizabeth D. Williams, Michael Henderson, Erik W. Thompson, Kara L. Britt

**Affiliations:** 1 Department of Surgery, University of Melbourne, St Vincent’s Hospital, Melbourne, VIC 3156 Australia; 2 St Vincent’s Institute of Medical Research, Melbourne, VIC 3156 Australia; 3 Department of Pathology, Peter MacCallum Cancer Centre, 305 Grattan St, Melbourne, VIC 3000 Australia; 4 Department of Pathology, University of Melbourne, Grattan Street, Parkville, VIC 3010 Australia; 5 Sir Peter MacCallum Department of Oncology, University of Melbourne, Grattan Street, Parkville, VIC 3010 Australia; 6 Department of Pathology, St Vincent’s Hospital, Melbourne, VIC 3156 Australia; 7 Austin Health and Northern Health, Melbourne, VIC 3084 Australia; 8 College of Health and Biomedicine, Victoria University, St Albans, VIC 8001 Australia; 9 Department of Biochemistry and Molecular Biology, School of Biomedical Sciences, Monash University, Clayton, VIC 3800 Australia; 10 Australian Institute for Musculoskeletal Science (AIMSS), Victoria University, University of Melbourne and Western Health, Sunshine Hospital, St Albans, VIC 3021 Australia; 11 Institute of Health and Biomedical Innovation and School of Biomedical Sciences, Queensland University of Technology, 2 George Street, Brisbane, QLD 4001 Australia; 12 Translational Research Institute, 37 Kent Street, Woolloongabba, QLD 4102 Australia; 13 Australian Prostate Cancer Centre – Queensland, Brisbane, QLD 4102 Australia; 14 Division of Surgery, Peter MacCallum Cancer Centre, Melbourne, VIC 3002 Australia; 15 Department of Anatomy and Developmental Biology, Monash University, Melbourne, VIC 3800 Australia; 16 Metastasis Research Laboratory, Peter MacCallum Cancer Centre, Melbourne, Victoria 3000 Australia

**Keywords:** Breast cancer, MCF10DCIS.com, Mammographic density, Murine biochamber

## Abstract

**Background:**

High mammographic density (HMD) not only confers a significantly increased risk of breast cancer (BC) but also is associated with BCs of more advanced stages. However, it is unclear whether BC progression and metastasis are stimulated by HMD. We investigated whether patient-derived HMD breast tissue could stimulate the progression of MCF10DCIS.com cells compared with patient-matched low mammographic density (LMD) tissue.

**Methods:**

Sterile breast specimens were obtained immediately after prophylactic mastectomy from high-risk women (*n* = 10). HMD and LMD regions of each specimen were resected under radiological guidance. Human MCF10DCIS.com cells, a model of ductal carcinoma in situ (DCIS), were implanted into silicone biochambers in the groins of severe combined immunodeficiency mice, either alone or with matched LMD or HMD tissue (1:1), and maintained for 6 weeks. We assessed biochamber weight as a measure of primary tumour growth, histological grade of the biochamber material, circulating tumour cells and metastatic burden by luciferase and histology. All statistical tests were two-sided.

**Results:**

HMD breast tissue led to increased primary tumour take, increased biochamber weight and increased proportions of high-grade DCIS and grade 3 invasive BCs compared with LMD. This correlated with an increased metastatic burden in the mice co-implanted with HMD tissue.

**Conclusions:**

Our study is the first to explore the direct effect of HMD and LMD human breast tissue on the progression and dissemination of BC cells in vivo. The results suggest that HMD status should be a consideration in decision-making for management of patients with DCIS lesions.

**Electronic supplementary material:**

The online version of this article (doi:10.1186/s13058-016-0767-4) contains supplementary material, which is available to authorized users.

## Background

*Mammographic density* (MD) refers to the radio-opaque tissue on a mammogram. High mammographic density (HMD) is associated with a higher rate of breast cancer (BC). Indeed, women in the highest MD quartile have a four to six times increased risk of BC compared with the lowest quartile after adjustment for age and body mass index (BMI), a relative risk that is second only to *BRCA1/2* gene mutation [1, 2]. It is not clear why HMD is associated with this increased BC risk, although reduced MD has been associated with response to hormone therapy in both prevention and treatment settings, as reviewed by Huo et al. [3, 4]. HMD is not uncommon; 42 % of women in the 40- to 59-year-old age group and 25 % of women in the 60- to 79-year-old age group have breasts that are at least 50 % mammographically dense [5].

Ursin and colleagues retrospectively assessed mammograms taken prior to and at the time of ductal carcinoma in situ (DCIS) diagnosis. They found that DCIS lesions occurred primarily in areas of HMD, suggesting that MD may stimulate BC initiation [6]. BCs that arise within areas of HMD are more commonly associated with factors indicative of a poor prognosis, including large tumour size, high histological grade, lymphovascular invasion and advanced stage, as compared with those arising within low mammographic density (LMD) [7–9]. It is not clear whether HMD increases the risk of metastasis. Two studies have shown that HMD is associated with an increased rate of local recurrence after breast-conserving surgery, but not with distant recurrence [10, 11]. We found that cytokeratin (CK)-positive tumour cells in HMD connective tissue are associated with local recurrence but not with distant metastasis [12]. Also, we discovered that collagen matrices representing concentrations of HMD seen in ductal carcinoma tissues induced increased BC cell migration compared with LMD tissue [13]. Increased stromal collagen in mouse mammary tissue was also shown to result in more invasive tumour phenotypes [14].

In order to assess whether HMD has any causal relationship with BC risk, we developed a biochamber mouse model that can viably grow and maintain the MD differential of normal breast tissue [15]. In the present study, we used it to determine whether HMD could stimulate the progression of DCIS-like lesions.

## Methods

### Sample accrual

This study was approved by the Peter MacCallum Human Research Ethics Committee (08/21) and St Vincent’s Hospital Melbourne Animal Ethics Committee (09/14). Between 2014 and 2015, ten women undergoing prophylactic mastectomy at St Vincent’s Hospital Melbourne provided consent through the Victorian Cancer Biobank. All participants gave their written informed consent for tissue accrual and publication of the study results. These women underwent the prophylactic procedure because of confirmed gene mutation carrier status and/or a strong family or past history of BC. Women were excluded from the study if suspicious lesions were visualised by pre-operative imaging.

### Tissue handling and selection of high and low mammographic density regions

Tissue sampling was carried out as previously described [15–18]. In brief, immediately after mastectomy, a 1-cm slice of breast tissue was resected from the fresh mastectomy specimen in a sterile environment by breast pathologists. HMD and LMD regions of the tissue slice were identified by examining specimen radiograms. Selected HMD and LMD tissues were then separately minced with a scalpel and mixed 1:1 with BD Matrigel™ (BD Biosciences, Billerica, MA, USA) supplemented with basic fibroblast growth factor (1 μg/ml; Sigma-Aldrich, Sydney, Australia) using sterile technique [16].

### Preparation for in vivo monitoring of MCF10DCIS.com cells

MCF10DCIS.com (DCIS.com) cells were provided by Robert J. Pauley, Barbara Ann Karmanos Cancer Institute, Detroit, MI, USA [19]. Luciferase/mCherry tagging [20] of DCIS.com cells is described in Additional file 1: Supplementary Methods. Cells were maintained in DMEM/F-12 medium (1:1) supplemented with 5 % horse serum and 4 mM glutamine [21, 22] in a humidified incubator (37 °C/5 % CO_2_). The top 10 % of mCherry-expressing cells were selected using flow cytometry and propagated in vitro for a maximum of two passages in preparation for murine chamber implantation, with or without fresh human mammary tissue.

### Murine xenograft model and ex vivo analysis of tumour burden using bioluminescence

For all experiments, mouse care was carried out in accordance with St Vincent’s Animal Ethics Committee guidelines. Patient-paired HMD or LMD breast tissue was mixed with 1 × 10^5^ DCIS.com cells and then suspended in Matrigel™ before being placed in the silicone chamber in the right groin of 6 week-old female severe combined immunodeficiency mice (*n* = 4 for HMD/woman, 4 for LMD/woman, 40 μl per chamber) vascularised by the inferior epigastric pedicle, as described previously [15, 17, 18]. As controls, 1 × 10^5^ DCIS.com cells were also inserted into biochambers with Matrigel™ without ﻿prior mixing with any human breast tissue﻿ in four separate mice each time a patient sample was processed (i.e., *n* = 4/woman) (see Additional file 2: Figure S1).

Tissue material from the biochambers was harvested at 6 weeks. Mice were given an intraperitoneal injection of 150 mg/kg luciferin (Promega, Madison, WI, USA), placed under anaesthesia using inhaled isoflurane, had blood drawn via cardiac puncture and then were humanely killed after 10 minutes. Biochamber material and mouse organs were removed and imaged ex vivo. Further details on luciferase and mCherry/red fluorescent protein (RFP) imaging are provided in Additional file 1: Supplementary Methods. Following imaging, the harvested materials were immediately fixed in 10 % neutral buffered formalin for 24 h before being transferred to 70 % ethanol for storage. Samples were subsequently processed, embedded in paraffin and sectioned at 5-μm thickness for histological and immunohistochemical analyses.

### Circulating tumour cell processing and quantification

Mouse blood was processed with red blood cell lysis buffer and PBS to remove serum and bulk erythrocytes. Further details on circulating tumour cell (CTC) processing are provided in Additional file 1: Supplementary Methods. The resulting pellets were then mixed with 500 μl of DCIS.com cell culture medium, and 400 μl of the suspension were plated in 60 × 15-mm cell culture dishes (CELLSTAR®; Greiner Bio-One GmbH, Frickenhausen, Germany) along with additional 1.5 ml of DCIS.com culture medium. This mixture was maintained in culture for 7 days. Plates were washed with PBS prior to crystal violet staining and inspection for mCherry-positive CTCs. Blood from naïve mice (*n* = 7) was also collected, processed and cultured as a negative control. Fluorescence microscopy was performed (Zeiss Axio Vert.A1; Carl Zeiss Microscopy, Thornwood, NY, USA) to identify mCherry-tagged DCIS.com cells. A bright-field Leica DFC425 microscope (Leica Microsystems, Buffalo Grove, IL, USA) was then used to image four random areas of each plate at × 20 magnification for manual counting and comparison of CTCs. CTCs were identified using bright field microscopy on the basis of their size and morphology that were evident under the fluorescence microscope.

### Assessment of harvested chamber tissue

Haematoxylin and eosin (H&E) staining was performed with all biochamber explants (harvested chamber tissue) and organ specimens in which positive luciferase and fluorescent signals were detected. A consultant pathologist (CK) who was blinded to the experimental groups assessed the H&E-stained slides of all chamber explants to determine the presence or absence of DCIS and invasive ductal carcinoma (IDC). Invasive carcinomas, when present, were scored for glandular differentiation, nuclear pleomorphism and mitotic count using the Nottingham grading system [23, 24].

### Immunohistochemical staining

The presence of DCIS versus IDC was further confirmed on representative slides with myoepithelial marker human-specific p63 immunohistochemical nuclear staining (Dako M7247, clone 4A4; Dako, Carpinteria, CA, USA) [25, 26]. Numerical categories were assigned to the histological results for comparisons among the three groups. These details are given in Additional file 1: Supplementary Methods. Tissue sections were photographed at × 10 and × 20 magnification using an AxioVision microscope (Carl Zeiss Microscopy).

To confirm metastases in luciferase-positive organs that did not show clear evidence of cancer on H&E stains, we sectioned the entire paraffin tissue block and stained consecutive sections with human-specific antibodies against cytokeratin 5 (CK5) (NCL-CK5, clone XM26; Leica Biosystems, Buffalo Grove, IL, USA) [27, 28] and DNA repair protein Ku70 (ab58150; Abcam, Cambridge, MA, USA) [29, 30]. Details regarding Ku70 and CK5 staining are provided in Additional file 1: Supplementary Methods.

### Statistical analyses

For each of the ten women, four mice were used for each of the DCIS.com + HMD, DCIS.com + LMD and DCIS.com-only groups. The mean value of all four mice from each experimental group was calculated and used as a representative value for that woman. HMD + DCIS.com, LMD + DCIS.com, and DCIS.com-only groups were compared using patient-matched (paired) one-way analysis of variance and Tukey’s multiple comparisons test using GraphPad Prism® version 6.00 for Windows software (GraphPad Software, La Jolla, CA, USA). Prior to all analyses, normality tests were used to confirm whether parametric tests were appropriate. The Grubbs test was used to detect any significant outliers for each set of data before it was analysed. All statistical tests were two-sided. Error bars in all graphs indicate SEM, and a *P* value <0.05 was considered to be statistically significant.

## Results

### Demographic characteristics of study participants

Our cohort of mammary tissue donors comprised a group of pre-menopausal and post-menopausal women with a mean age of 45 years. Six of them had confirmed gene mutation carrier status. The other four women underwent prophylactic mastectomy because of a significant past or family history of BC. Breast Imaging-Reporting and Data System (BI-RADS) scores ranged from 1 to 4 across the cohort. The demographic characteristics of the cohort are summarised in Table 1.

**Table 1:** Demographic characteristic of study participants (*n* = 10)

Selected characteristics	Number or mean
Age at surgery date	Mean 45 years, median 43 years, range 31–64 years
BI-RADS score, *n*	
4	2
3	2
2	4
1	2
Risk factors (some women had more than one risk factor)	
Strong family history	6
*BRCA1* mutation-positive	2
*BRCA2* mutation-positive	3
*PTEN* mutation-positive	1
Past history of BC or DCIS	7
Menopausal status	
Pre-menopausal	5
Peri-menopausal	2
Post-menopausal	3
Parity	
Parous	8
Nulliparous	2

*Abbreviations: BI-RADS* Breast Imaging-Reporting and Data System, *BC* Breast cancer, *IDC* Invasive ductal carcinoma, *DCIS* Ductal carcinoma in situ

BI-RADS score 1 = predominantly fat, 2 = scattered fibroglandular densities, 3 = heterogeneously dense, 4 = extremely dense

### Analyses of weights and histopathology of chamber explant materials

**Fig. 1:**
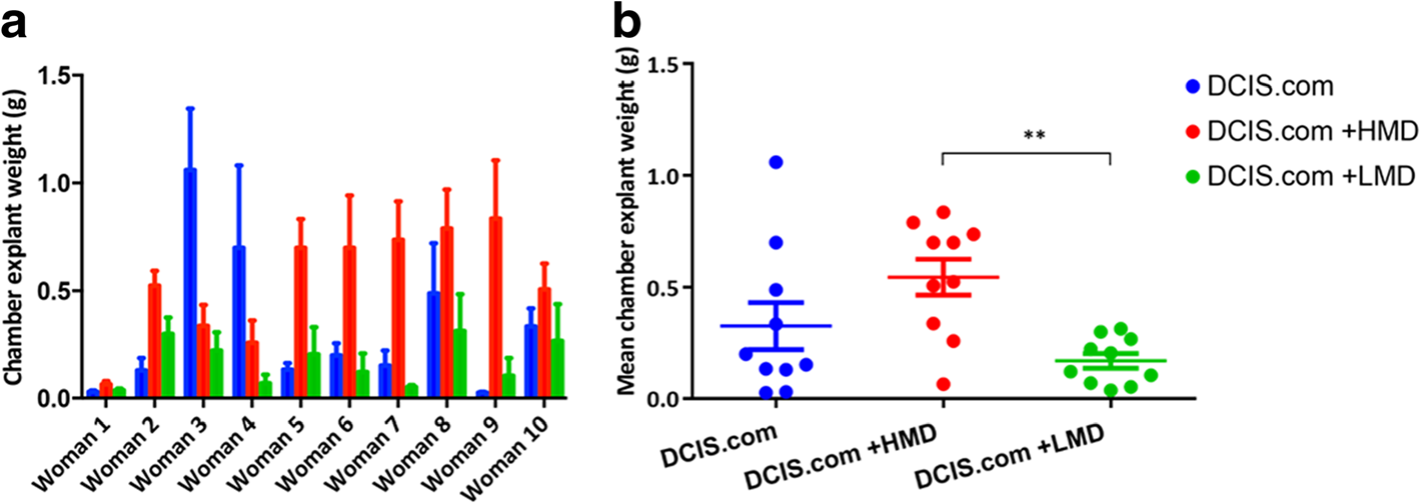
Quantitative analyses of chamber explant weights. **a** Coloured bar graphs show patient-matched DCIS-only (*blue*), DCIS + HMD (*red*) and DCIS + LMD (*green*) comparisons of chamber explant weights for all ten women. **b** Average values for each woman are shown as scatterplots. *HMD* High mammographic density, *LMD* Low mammographic density, *DCIS* MCF10DCIS.com cells. **P = 0.002. All bar and scatterplot graphs represent mean + SEM

The mean weights of biochamber materials from the HMD + DCIS.com group (*n* = 4 mice/patient) were on average two times greater than those in the DCIS.com-only (*n* = 4 mice/patient) and DCIS + LMD (*n* = 4 mice/patient) groups (Fig. 1). Biochamber weights from the HMD + DCIS.com group were significantly greater than those in the LMD + DCIS.com group (*P* = 0.002), but they were not significantly greater than those in the DCIS.com-only group. Explant weights from the LMD + DCIS.com group tended to be less than those in the DCIS.com-only group, but this difference was not statistically significant (Fig. 1).

**Fig. 2:**
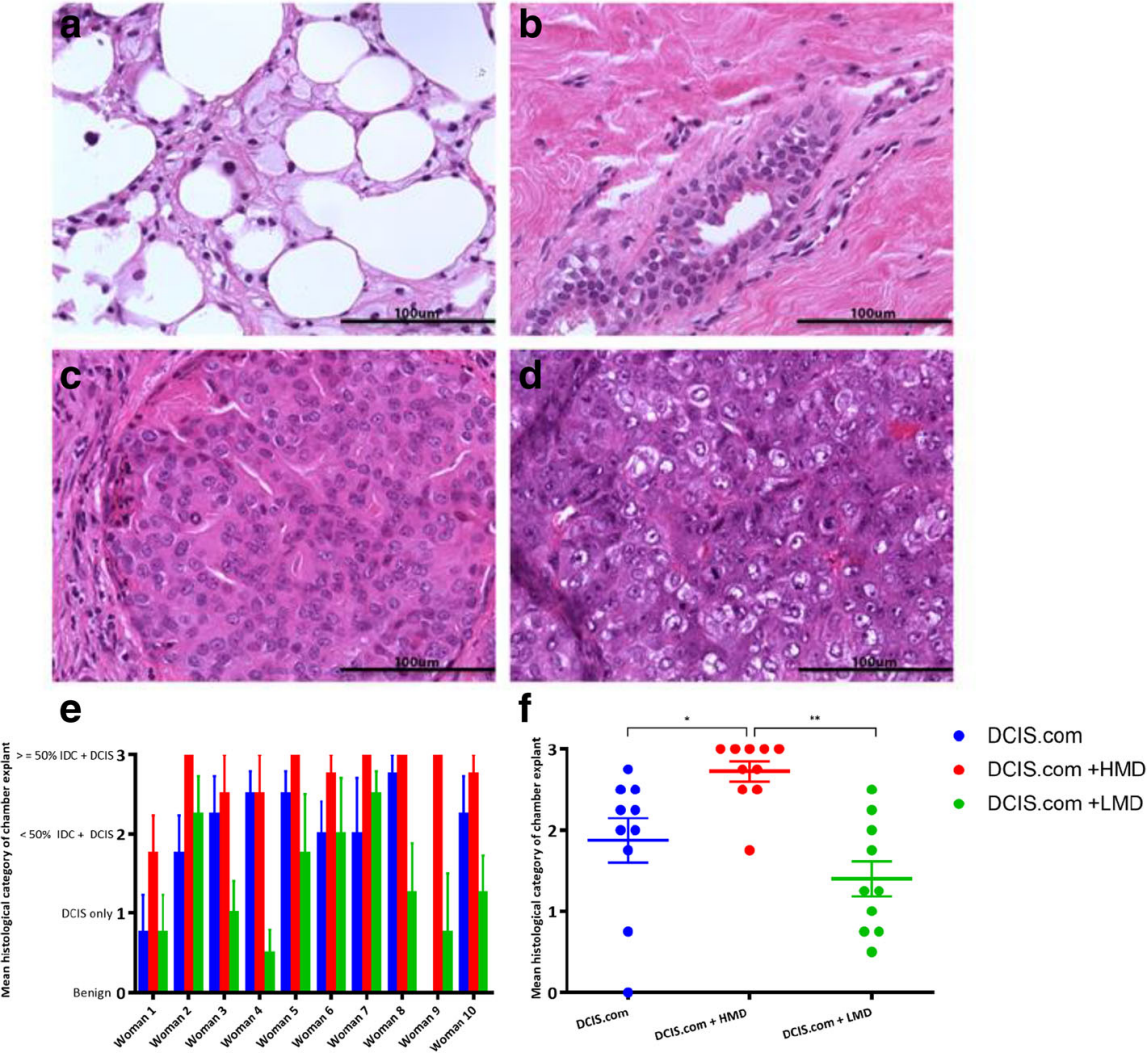
Quantitative analysis of the histopathological results of chamber material. **a**–**d** Representative photomicrographs of the various numerical categories used to score human breast histopathology observed in biochambers. **a** Category 0, the absence of DCIS and cancer and the presence of fatty normal breast tissue. **b** Category 0, the absence of DCIS and cancer and the presence of dense normal breast tissue. **c** Category 1, high-grade DCIS only. **d** Category 2-3, grade 3 invasive carcinoma. **e** The numerical histological categories 0–3 included a breakdown of the presence of high-grade DCIS alone or along with a certain percentage of grade 3 IDC. The mean histological category for the chamber explants for each of the ten women according to the type of input material. **f** Average values for each woman for the histological category of the chamber material. *HMD* High mammographic density, *LMD* Low mammographic density, *DCIS* MCF10DCIS.com cells, *IDC* Invasive ductal carcinoma. *P = 0.004; **P = 0.002. All bar and scatterplot graphs represent mean ± SEM

The histology of the biochamber materials contained benign mammary tissue, high-grade DCIS lesions only or high-grade DCIS with grade 3 IDC (Fig. 2a–d). p63 staining showed that the myoepithelial layer remained intact in normal mammary ducts as well as in DCIS lesions; however, this integrity was lost in IDC, despite tumour cells staining positive with p63 because of their basal phenotype (see Additional file 3: Figure S2). The percentage of high-grade DCIS with grade 3 IDC in the HMD + DCIS.com group was higher than in both the DCIS.com-only and LMD + DCIS.com groups for nine of ten women (Fig. 2e). When we compared the mean values of all ten women, we observed that the HMD + DCIS.com group had a significantly higher proportion of high-grade DCIS and grade 3 IDC than the other groups (Fig. 2f). In the presence of LMD tissue, the resulting percentage of high-grade DCIS and grade 3 IDC was often lower than in the DCIS.com-only group; however, this trend was not statistically significant.

### Luciferase signal and tumour burden

**Fig. 3:**
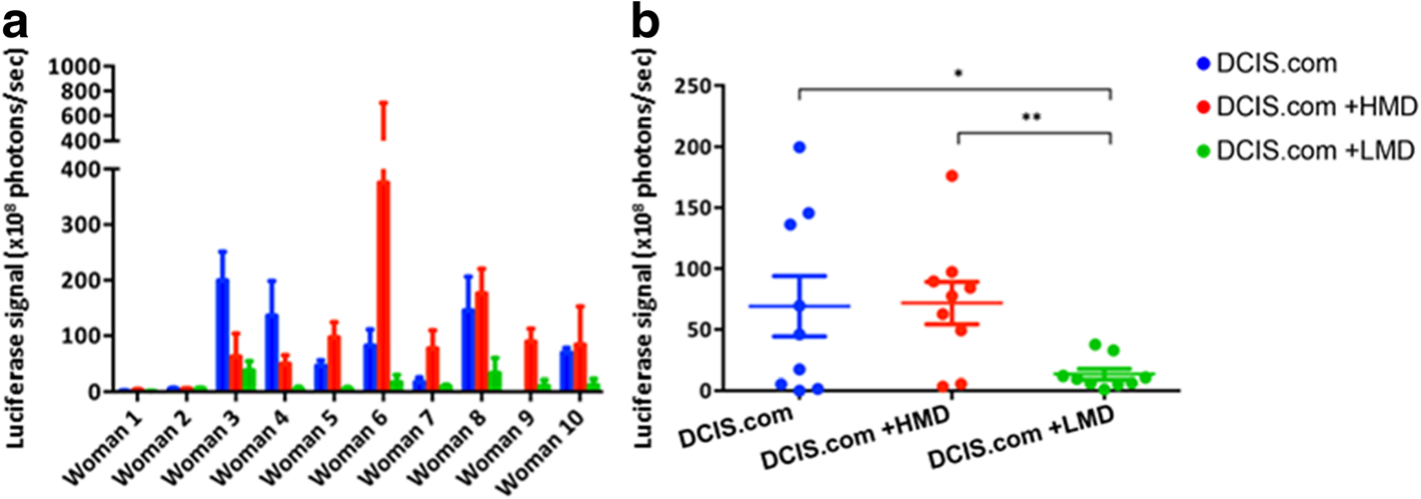
Analyses of chamber explant luciferase signalling. **a** Luciferase signal measured in the chambers at explant (×10^8^ photons per second) for each of the ten women. **b** Average values for each woman are shown as scatterplots after removal of a significant outlier (patient 6). *HMD* High mammographic density, *LMD* Low mammographic density, *DCIS* MCF10DCIS.com cells. *P = 0.03; **P = 0.005. All bar and scatterplot graphs represent mean ± SEM

Both luciferase and mCherry images were collected for the first three accruals, and the results were comparable (Additional file 4: Figure S3). Hence, only bioluminescence images were used for analyses thereafter. HMD + DCIS.com biochambers showed significantly higher bioluminescent signalling than the LMD + DCIS.com group (*P* = 0.005). Interestingly, the LMD + DCIS.com group also showed a significant reduction in bioluminescent signalling compared with the DCIS.com-only group (*P* = 0.03) (Fig. 3).

### Comparison of ‘take’ rates of MCF10DCIS.com cells in biochambers

**Fig. 4:**
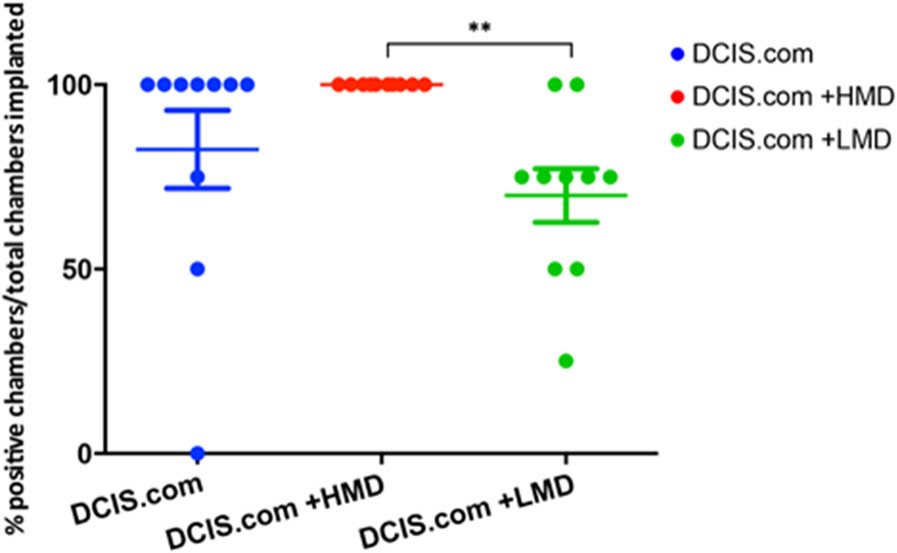
Comparison of chamber ‘take’ rates representing the viability of input MCF10DCIS.com cells. The average number of positive biochambers (contained a histological category of at least 1: DCIS material only) as a percentage of the total number of chambers implanted. *HMD* High mammographic density, *LMD* Low mammographic density, *DCIS* MCF10DCIS.com cells. **P = 0.008. Error bar indicates SEM

For each woman, the ‘take’ rate was assessed as the proportion of mice in each experimental sub-group (*n* = 4) that had a positive DCIS.com presence after 6 weeks as confirmed by IVIS in vivo imaging and histology (PerkinElmer, Waltham, MA, USA). The HMD + DCIS.com group was the only group to have a 100 % take rate, while the DCIS.com-only and LMD + DCIS.com groups had 82.5 % and 70.0 % take rates, respectively (*n* = 10 women) (Fig. 4).

### Comparison of tumour metastasis in DCIS.com + HMD, DCIS.com + LMD and DCIS.com-only groups

**Fig. 5:**
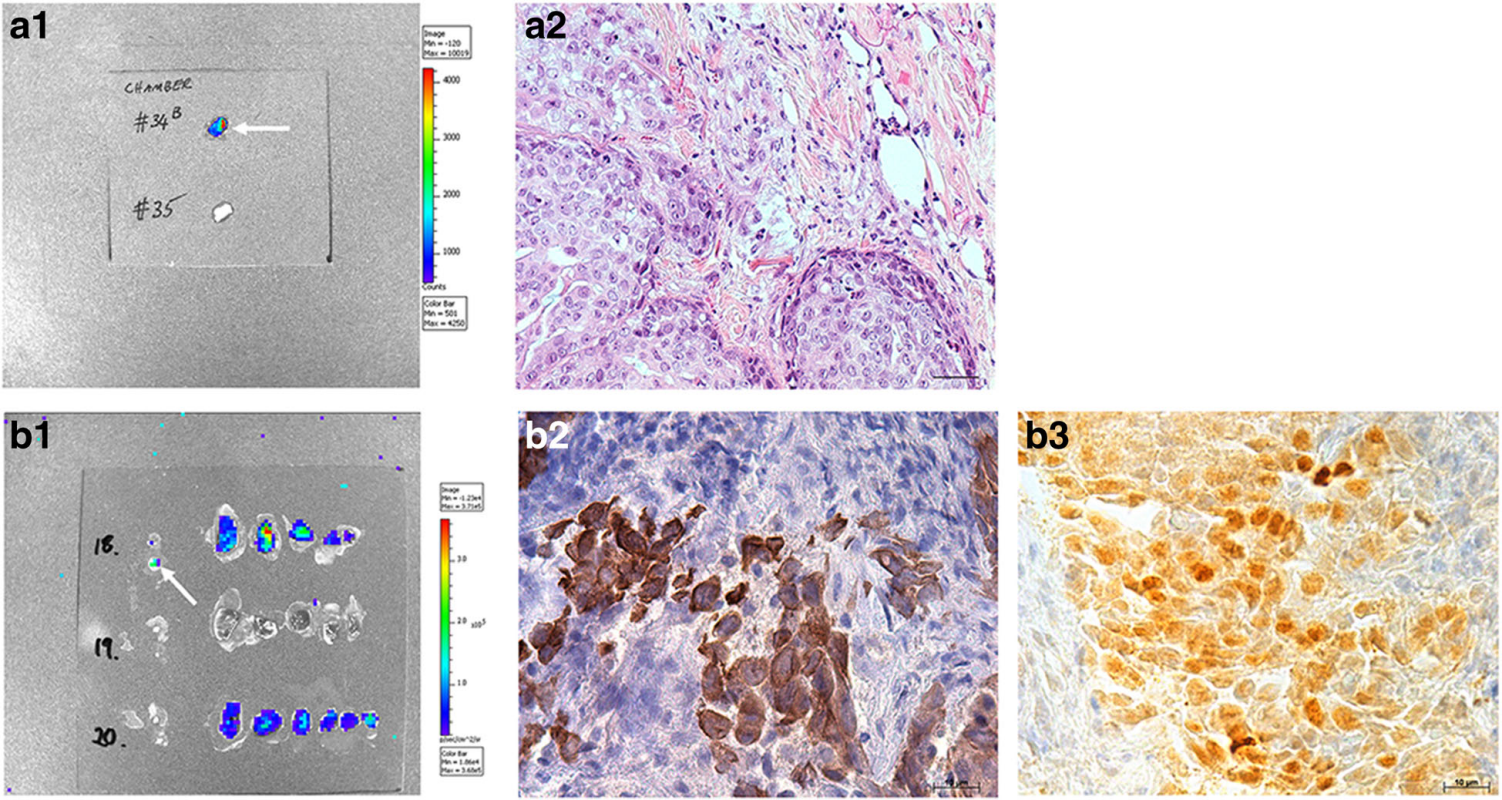
Representative images of tumour metastasis obtained by imaging and histology. **a1** and **a2** Images from the same mouse lymph node. **b1**–**b3** Images illustrate the lymph node of a different mouse. **a1** The *white arrow* indicates a luciferase (luc) signal in a mouse lymph node. **a2** Haematoxylin and eosin staining of the luc-positive lymph node from **a1**. **b1** The *white arrow* indicates a luc signal in a mouse lymph node. **b2** Human-specific CK5 staining of the luc-positive lymph node from **b1**; CK5-positive cells are stained *brown*. **b3** Human-specific Ku70 staining of the luc-positive lymph node from **b1**. Ku70-positive cells are stained *yellow*. Scale bar = 10 μm

Cancer metastases were detected by ex vivo bioluminescence imaging most frequently in axillary lymph nodes and lungs, and occasionally in the liver and/or bowel. Areas with positive luciferase signals were dissected and stained with H&E for histological confirmation of metastasis (Fig. 5a1, a2). Occasionally, small clusters of cancer cells were visualised by imaging (*arrow* in Fig. 5b1), but they were not readily identified upon H&E staining. In those cases, human-specific CK5 and Ku70 staining confirmed the presence of tumour cells of human origin (Fig. 5b1–b3).

**Fig. 6:**
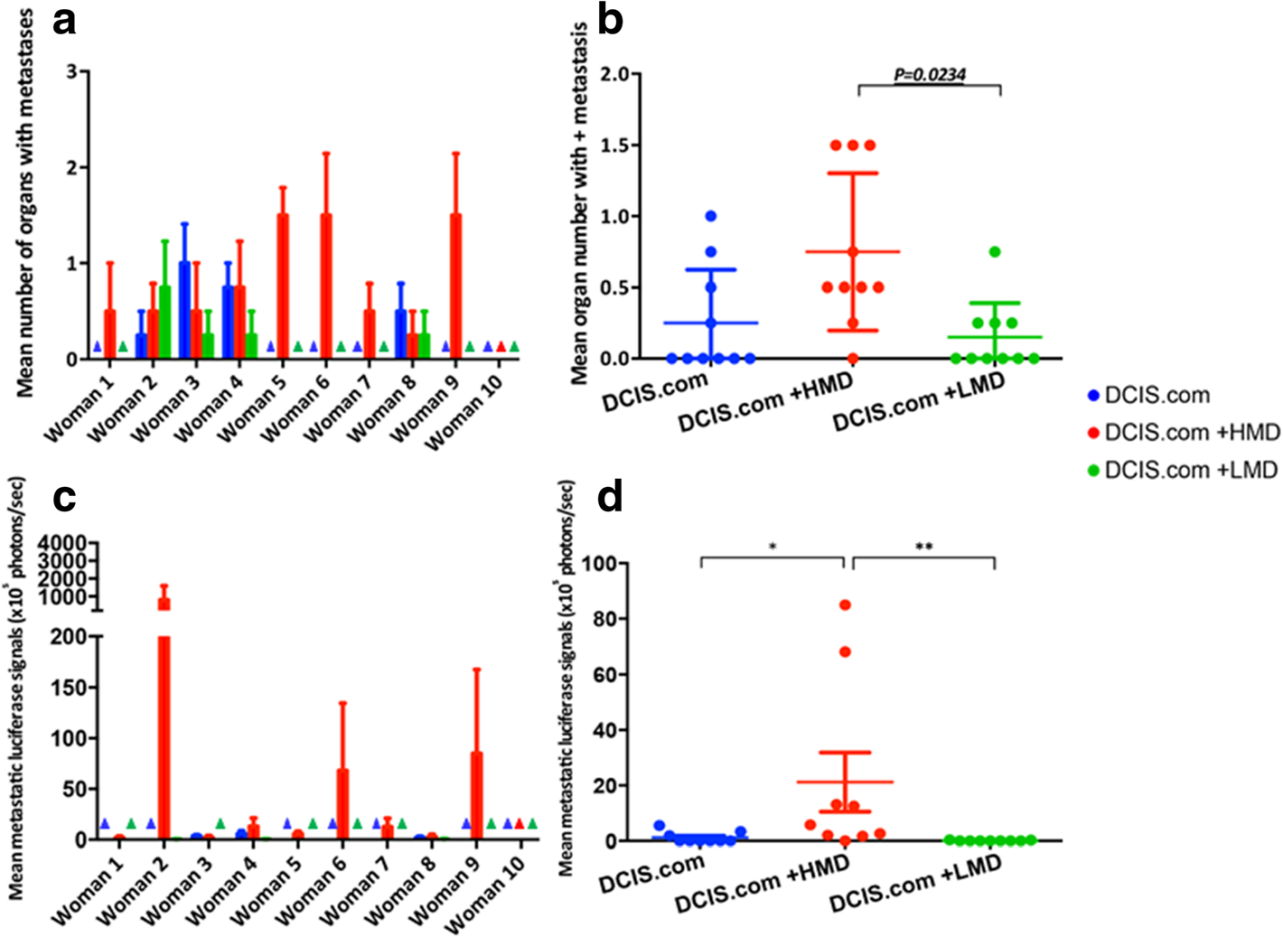
Quantitative analyses of metastases in terms of metastasis-positive organ numbers and luciferase signalling. **a** Coloured bar graphs of the mean value of all four mice for each group for mean metastasis + organ numbers. **b** Averaged values of the mean number of metastasis-positive organs for each woman according to the type of input material. **c** Coloured bar graphs of the mean level of luciferase in metastasis-positive organs (×10^5^ photons/second). **d** Averaged values of the mean luciferase load in metastasis for each woman. *HMD* High mammographic density, *LMD* Low mammographic density, *DCIS* MCF10DCIS.com cells. *P = 0.02; **P = 0.008. All bar and scatterplot graphs represent mean ± SEM; *triangles* indicate data value of 0

**Fig. 7:**
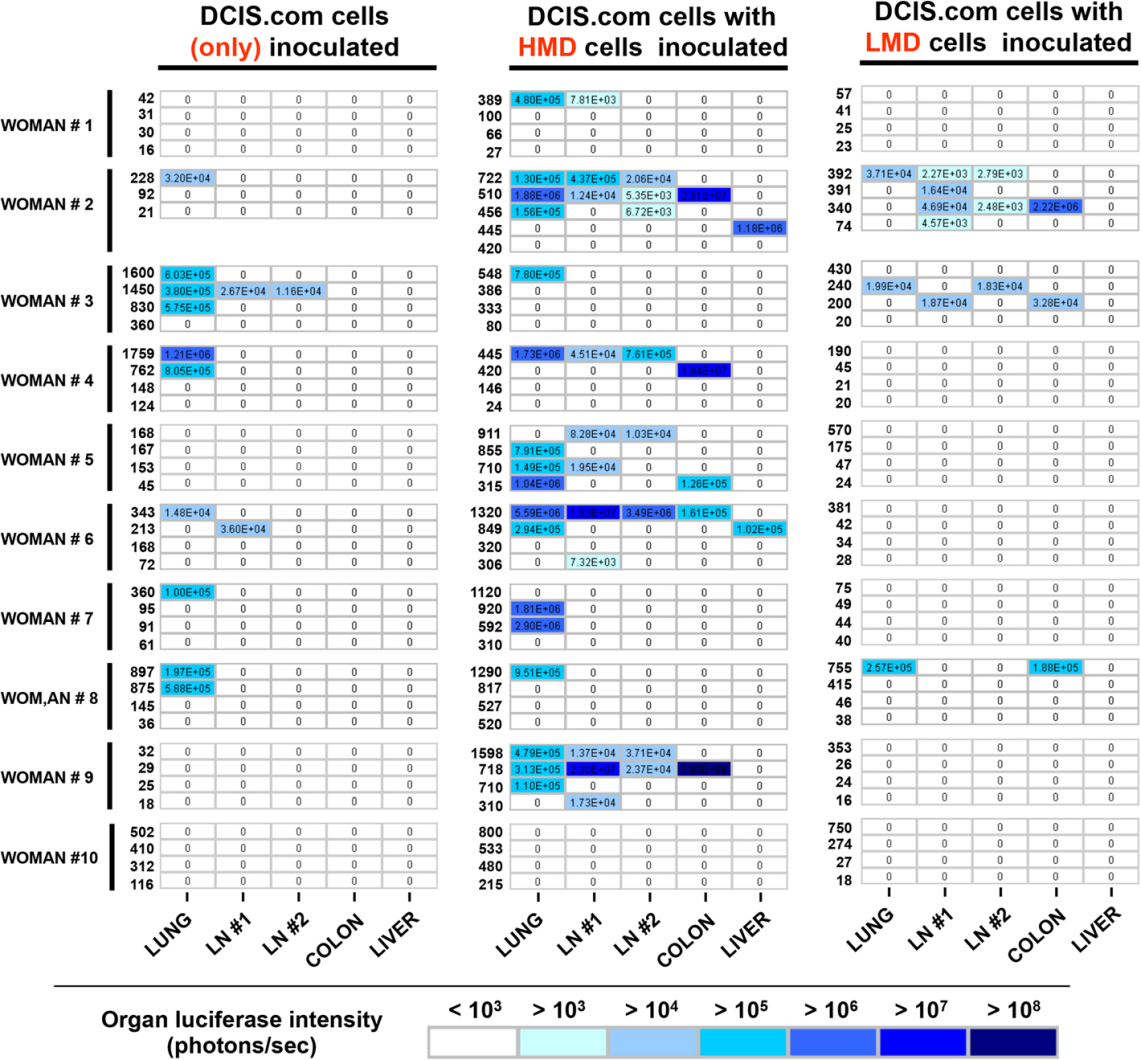
Heat map of metastasis profile for each woman. The degree of metastasis is depicted using *filled blue squares* to denote metastasis-positive organs, and the level of metastatic burden is shown using the colour code shown. Patients 1–10 are shown in rows. For each woman, four mice were implanted with DCIS alone, four with DICS + HMD and four with DCIS + LMD. The grouped columns represent the types of cells implanted, and the sub-columns represent the site of metastatic burden. The numbers in *boldface* type at the beginning of each row represent the chamber explant weight to show the correlation between chamber weight and metastasis. Inside each bar, the luciferase levels in photons per second are indicated

The number of organs with cancer metastases was counted per mouse for each group (Fig. 6a). The HMD + DCIS.com group had significantly higher mean numbers of metastasis-positive organs than the LMD + DCIS.com group across all ten women (*P* = 0.0234) (Fig. 6b). The mean quantities of bioluminescent signalling in metastases were significantly increased in the HMD + DCIS.com compared with the LMD + DCIS.com group (*P* = 0.008) (Fig. 6c and d). As an alternate representation of the data, the heat map shown in Fig. 7 depicts that mice implanted with HMD + DCIS.com tissue had higher frequencies and amounts of distal metastatic burden.

**Fig. 8:**
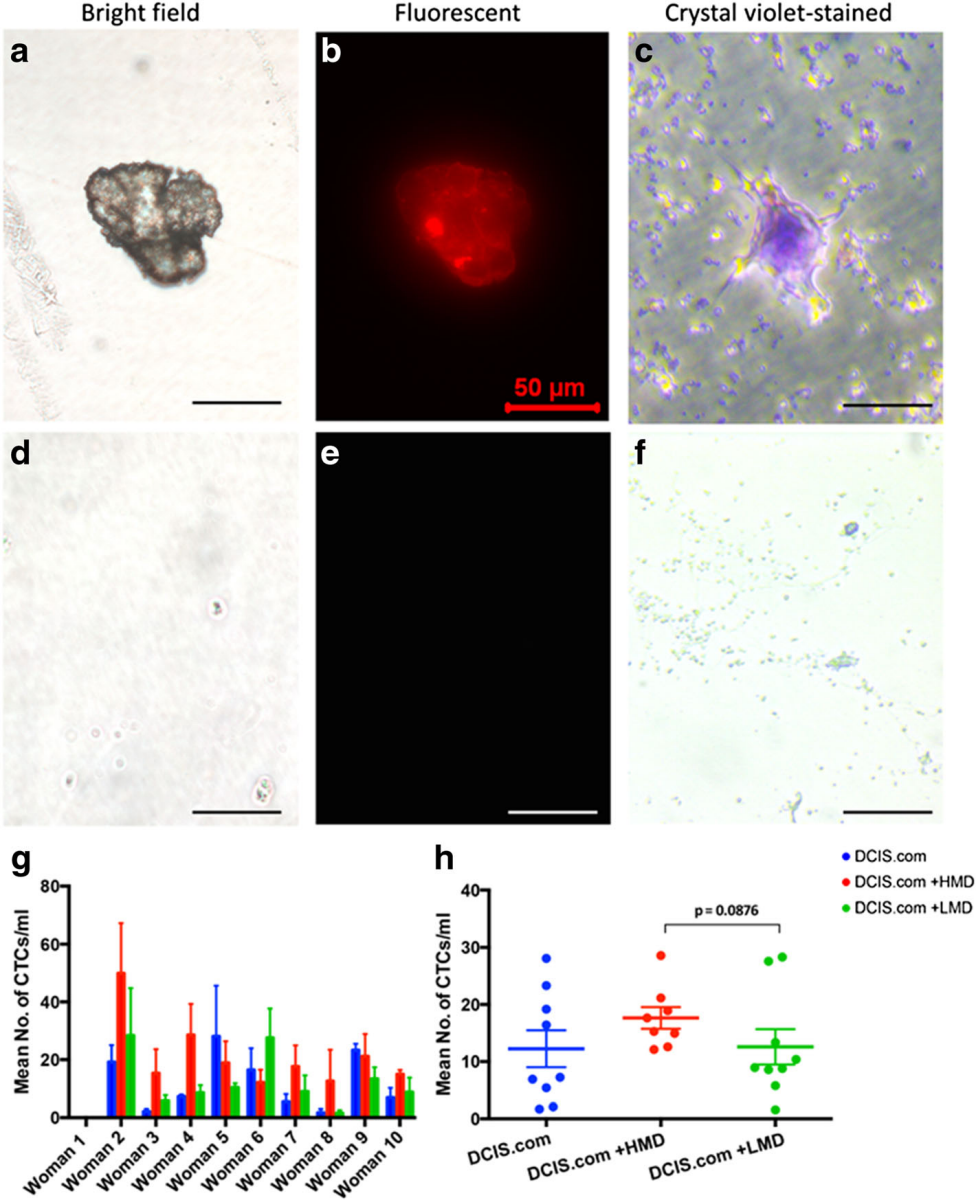
CTC analyses. **a** Representative photomicrographs under bright field of CTCs isolated and cultured blood from mice blood. **b** Corresponding fluorescence image to demonstrate RFP tagging in CTCs. **c** Representative photomicrographs of CTCs after crystal violet staining. **d**–**f** As negative controls, blood taken from naïve mice was also processed and cultured, and images were obtained to show the absence of CTCs. **g** Coloured bar graphs of the mean value of all four mice for each group for CTC numbers. **h** Averaged values of the mean number of CTCs for each woman according to the type of input material. *CTC* Circulating tumour cell, *HMD* High mammographic density, *LMD* Low mammographic density, *DCIS* MCF10DCIS.com cells, *RFP* Red fluorescent protein. All bar and scatterplot graphs represent mean ± SEM. Scale bar = 50 μm

To assess the levels of CTCs, mouse blood was collected from nine of the ten independent experiments and cultured for 7 days. CTCs of approximately 50 μm in diameter that were mCherry-positive and stained with crystal violet were detected in the blood samples from all nine accruals (Fig. 8). By contrast, there were no similarly sized colonies present in cultures generated from tumour-naïve mice. These cultures also lacked mCherry signalling and crystal violet staining. The number of CTCs per mouse was counted and adjusted for the volume of blood collected. When averaged per patient, the number of CTCs per millilitre was not significantly increased in the HMD + DCIS.com group compared with the LMD + DCIS.com group (*P* = 0.0876) (Fig. 8).

### Correlation between participant demographics and DCIS.com cellular responses

When assessing the chamber responses of patient-matched HMD + DCIS.com, LMD + DCIS.com and DCIS.com-alone groups according to individual women’s demographic characteristics (see Additional file 5: Figure S4), we found that the results from post-menopausal women (patients 3, 4 and 7, all of whom were parous and known to be gene mutation-negative and had overall non-dense breasts [BI-RADS scores 2 and 1]) did not differ from those of pre-menopausal women (*n* = 7) in terms of mean histological categories and CTCs. However, the mean chamber explant weight, luciferase signalling and metastases from HMD tissue of patient 3 were all lower than corresponding parameters of the DCIS.com-alone group, which may have been due to variations in the DCIS.com cells alone. These results from patient 7 followed the overall trend of the HMD group being associated with increased tumour growth. When we compared the chamber responses from breast tissues of gene mutation carrier-positive women (patients 1, 2, 4, 5, 9 and 10) with those of gene mutation-negative women (patients 3, 6, 7 and 8), and between women with overall dense breasts (BI-RADS score 3 or 4: patients 2, 6, 8 and 9) and women with overall non-dense breasts (BI-RADS score 1 or 2: patients 1, 3, 4, 5, 7 and 10), we found no significant differences. Furthermore, nulliparous women (patients 8 and 10) did not show a different trend with regard to chamber outgrowth weights or histological results. Both women had elevated numbers of CTCs in their HMD group, although no metastasis was found in mice for patient 10, and the number of metastasis-positive organs in the tissue groups of patient 8 was lower than that of the DCIS.com-alone arm (Additional file 5: Figure S4d–f). Overall, the finding of increased tumour growth and dissemination associated with the HMD + DCIS.com group as compared with the other two groups did not differ significantly by age, menopausal status, gene mutation carrier status, overall breast BI-RADS category or parity. Future studies with larger study cohorts are needed to provide sufficient power for assessing whether the pro-malignant effect of HMD is modulated by any of the aforementioned factors.

## Discussion

In this within-individual matched-sample study, we found that HMD breast tissue led to significantly increased tumour weight, greater proportions of high-grade DCIS and grade 3 IDCs, and metastasis of DCIS.com cells compared with LMD tissue from the same woman. The tumour-promoting effect of HMD was observed across women, despite the heterogeneous demographic characteristics of our cohort (mixed menopausal statuses and risk profiles). The finding of high-grade DCIS with grade 3 IDC was consistent with the characteristics of DCIS.com cells to form high-grade DCIS that progresses to correspondingly high-grade IDC in vivo [22]. To our knowledge, we are the first to demonstrate a causal relationship between HMD tissue and BC progression and metastasis in an in vivo setting. We also found a trend of increased CTCs in mice carrying biochambers implanted with HMD tissue compared with LMD and DCIS.com only; however, this was not statistically significant, which may be due to the variation in CTC numbers per mouse for each woman and between women, as well as to our small sample size.

There is little evidence on whether HMD directly affects the progression and metastasis of already established tumours; however, the pathobiology of HMD does support stimulated cancer progression [16, 31]. Boyd and colleagues found that HMD is associated with increased breast tissue stiffness [31], and HMD stroma has increased collagen organisation compared with LMD from the same woman [16, 32, 33]. Specifically, McConnell and colleagues found that increased collagen stiffness and organisation, not abundance, correlated with HMD in a cohort of 22 post-menopausal women (4 cancer-free and 18 with BC). In their study, the tissues from 18 women were sampled at least 4 cm away from tumours, albeit within the same breasts where cancer had initially arisen, and a total of 6 HMD samples were compared with 6 LMD tissue specimens of different women matched for age and menopausal status, but not for BMI or other confounding factors [32]. Their findings contrast with our earlier association of HMD with increased stroma and collagen content [16], where paired HMD breast tissue showed increased collagen organisation as well as abundance compared with LMD tissue of the same woman in a group of 15 cancer-free women. As McConnell et al. used Picrosirius red staining and atomic force microscopy, whereas we used second harmonic generation imaging coupled with grey-level co-occurrence matrix analysis; variable methodologies may also contribute to the differences in the results. Collagen of altered alignment, through mechanical and other unknown properties, have been shown to facilitate tumour growth [14, 34, 35]. The breast stroma is a rich source of numerous cell types, including fibroblasts, adipocytes and extracellular matrix (ECM) proteins [36–38]. ECM comprises not only collagen but also fibronectin, proteoglycans and matrix metalloproteinase (MMP) inhibitors, which have also been shown to enhance collagen stiffness and regulate growth factors and susceptibility to BC [3, 39–43]. Although BC is of epithelial cell origin and HMD is associated with increased benign epithelial lesions [44, 45], an increasing body of data supports the hypothesis that perturbations in stromal architecture are key to establishing a pro-neoplastic environment that enhances cancer growth [46, 47]. In addition, fibroblasts are a major stromal component and have been implicated in pro-malignant activity through the production and/or modification of cytokines, growth factors, ECM components and MMPs [48, 49].

Our results show that the incorporation of LMD breast tissue into DCIS.com cell inoculations reduced tumour weight, lowered the proportion of high-grade DCIS with grade 3 IDCs and led to less metastasis compared with incorporating HMD tissue, suggesting a protective role of the adipose-rich, dense, connective tissue-poor LMD tissue. Consistent with the trends and effects seen in our studies, the association of absolute dense area with BC risk was found to be decreased for larger breasts [50]. Investigators in two large case-control studies (634 cases:1880 controls [51] and 1424 cases:2660 controls [52]) and a prospective study of 111 cases of BC [53] all found statistically significant inverse associations of non-dense breast area with BC risk. There is limited evidence on how adipose tissue modifies BC risk and cancer progression. Fatty breast tissue secretes leptin, which was found to enhance BC cell proliferation, as well as adiponectin, which limits cell proliferation and promotes apoptosis of aberrant cells [54]. Whilst it is unknown whether adipose tissue produces more adiponectin than leptin, a balance between the two was proposed to alter BC risk [55]. Adipose tissue also stores vitamin D, known for its protective effect against cancer development through a wide range of roles, including cell-cycle arrest, apoptosis, repair and promotion of differentiation [56–58]. Little is currently known about cancer-associated adipose tissue, although it is known to secrete a range of cytokines (interleukin [IL]-6, IL-8, chemokine [C-C motif] ligand 5) and collagen VI, promoting BC progression and metastasis [59–63]. Further work on cytokines and ECM from HMD and LMD tissue is required to understand the relationship of the decreased adipose content in HMD and BC.

### Strengths and limitations

Previous work conducted by Chew and colleagues showed that human HMD and LMD breast tissues that were sampled from prophylactic mastectomy specimens, mechanically minced, mixed with Matrigel™ and then incubated in murine biochambers for 6 weeks remained viable, maintained their original histological characteristics and their MD status [15]. H&E, Masson’s trichrome blue and vimentin staining showed increased collagen and stromal content and a lower fat percentage in HMD chamber tissue compared with that in LMD, correlating with the histological composition of the original mastectomy specimens. Thus, we believe that mechanical mincing in preparation for chamber implantation and supplementation with Matrigel™ did not have a significant impact on the histological composition or collagen content of implanted tissues, and that any effect would be equally distributed to both HMD and LMD groups. In addition, we have unpublished work showing that, when the ECM structure is disrupted through collagenase, hyaluronidase and trypsin digestion of the samples, the original histology and MD status of the input material was not maintained in murine chambers [64]. Prior to chamber implantation, second harmonic generation imaging found that HMD breast tissue had a higher degree of stromal collagen organisation than LMD tissue [16]. However, future studies are needed to further assess whether mincing and addition of Matrigel™ would affect the mechanical stiffness, composition or actions of MMPs in the ECM of implanted breast tissue. Another potential limitation of our study is that our group of ten women was relatively young (mean age 45 years), and five of them were pre-menopausal. The results therefore cannot be directly extrapolated to the average population, in particular for post-menopausal women. However, studying normal breast tissue of different MDs enabled us to examine features of MD that may elevate BC risk prior to established tumour burdens in the breast. The within-individual study design also allowed us to compare HMD and LMD breast tissues of the same patient, eliminating all important confounding factors, such as age, BMI and menopausal status, which can be difficult to adjust for in across-patient studies.

## Conclusions

To the best of our knowledge, this  study is the first to demonstrate the direct effects of HMD and LMD human breast tissue on the growth and dissemination of BC cells in vivo. Further studies on stromal and ECM components will improve understanding of BC evolution and help to identify potential biological markers and therapeutic targets for more individualised management of patients with, or at high risk for, BC. Our data suggest a benefit of including the MD status in the assessment and therapeutic management of BC and DCIS.

## Additional files

Additional file 1: The murine xenograft model Schematic diagrams illustrate the use of 12 SCID mice associated with each woman’s tissue accrual and the allocation of 4 mice into HMD, LMD and DCIS.com only groups. HMD: high mammographic density; LMD: low mammographic density; DCIS.com: MFC10DCIS.COM cells. (DOCX 16kb)
Additional file 2: Figure S1. The murine xenograft model. Schematic diagrams illustrate the use of 12 SCID mice associated with each patient’s tissue accrual and the allocation of 4 mice into DCIS.com + HMD, DCIS.com + LMD and DCIS.com-only groups. The schematic mouse shows a silicone chamber inserted in the groin with chamber material (in *grey*) vascularised by the inferior epigastric pedicle (in *red*). *HMD* High mammographic density, *LMD* low mammographic density, *DCIS.com* MFC10DCIS.com cells. (TIF 537 kb)
Additional file 3: Figure S2. p63 immunohistochemical nuclear staining. **a** Representative photomicrograph of punctate brown nuclear staining with p63 of normal human mammary glands adjacent to invasive tumour cells from a chamber explant at × 10 original magnification. **b** Normal mammary ducts shown in (**a**) at × 20 original magnification. **c** Representative photomicrograph of punctate brown nuclear staining with p63 of a ductal carcinoma in situ lesion from a chamber explant at × 10 original magnification. **d** Ductal carcinoma in situ lesion shown in (**c**) at × 20 original magnification. **e** Representative photomicrograph of punctate brown nuclear staining with p63 of invasive ductal carcinoma adjacent to ductal carcinoma in situ lesions at × 10 original magnification. **f** Invasive ductal carcinoma cells adjacent to ductal carcinoma in situ lesions shown in (**e**) at × 20 original magnification. (TIF 7517 kb)
Additional file 4: Figure S3. Comparison of luciferase and mCherry imaging. **a** Representative luciferase imaging of three chamber explants. **b** Representative mCherry imaging of the same chamber explants shown in **a**. (TIF 2371 kb)
Additional file 5: Figure S4. Correlation between participant demographics and DCIS.com cellular responses. The table outlines the individual demographic characteristics of each patient (*n* = 10). **a** Coloured bar graphs show patient-matched DCIS.com only (*blue*), DCIS.com + HMD (*red*) and DCIS.com + LMD (*green*) comparisons of chamber explant weights (**a**), chamber explant luciferase signals (**b**), mean histological category of chamber explants (**c**), mean number of CTCs/ml (**d**), mean number of metastasis-positive mouse organs (**e**) and mean luciferase signals for total metastases (**f**) for each patient. *HMD* high mammographic density, *LMD* low mammographic density, *DCIS* MCF10DCIS.com cells, *CTC* circulating tumour cells, *BC* breast cancer, + positive, *N/A* Not available. *Triangles* indicate data value of 0. BI-RADS score 1 = predominantly fat, 2 = scattered fibroglandular densities, 3 = heterogeneously dense, 4 = extremely dense. (PPTX 500 kb)

## Abbreviations

BC: Breast cancer
BI-RADS: Breast Imaging-Reporting and Data System
CK: Cytokeratin
CTC: Circulating tumour cell
DCIS: Ductal carcinoma in situ
DCIS.com: MCF10DCIS.com cells
ECM: Extracellular matrix
H&E: Haematoxylin and eosin
HMD: High mammographic density
IDC: Invasive ductal carcinoma
IL: Interleukin
LMD: Low mammographic density
MD: Mammographic density
MMP: Matrix metalloproteinase
RFP: Red fluorescent protein
