# Supplementary material for: Mammographically dense human breast tissue stimulates MCF10DCIS.com progression to invasive lesions and metastasis
Source: Breast Cancer Res. 2016 Oct 25;18:106. doi: 10.1186/s13058-016-0767-4 (PMC5078949; doi:10.1186/s13058-016-0767-4)
Supplement: Additional file 1: — The murine xenograft model Schematic diagrams illustrate the use of 12 SCID mice associated with each woman’s tissue accrual and the allocation of 4 mice into HMD, LMD and DCIS.com only groups. HMD: high mammographic density; LMD: low mammographic density; DCIS.com: MFC10DCIS.COM cells. (DOCX 16kb) [file 13058_2016_767_MOESM1_ESM.docx]

**Supplementary Methods**

***Generation of luciferase tagged of MCF10DCIS.COM cells***

We used a retroviral bicistronic vector (pBabe luc2-IRES-mCherry) to generate DCIS.Com cells stably expressing modified firefly luciferase and monocherry. In brief HEK293 cells were transfected with 7 μg of Ampho vector and 7 μg pBABE luc2-IRES-mCherry as well as Lipofectamine PLUS reagent and LTX reagent according to the manufacturer’s instructions. Virus was harvested from these cells 2 days later and applied to DCIS.com cells in combination with polybrene (10ng/ml).

***The murine xenograft model and ex-vivo analysis of tumour burden using bioluminescence***

Harvested chamber explants were removed and then lungs, liver, bowels and bilateral axillary lymph nodes were all rapidly dissected. All harvested material was washed in PBS and transferred to 12-well plates and immersed in dilute luciferin solution (0.15mg/ml). Chamber explants and organs were imaged for luciferase and mCherry *ex-vivo* using an IVIS® Spectrum 2000 (Perkin Elmer, Waltham, MA) under consistent exposure and binning parameters. Luciferase signals (presented as photons/sec) were quantified in multiple orientations for each chamber explant and for every organ by manually demarcating the target of interest and subtracting appropriate background signal levels for the corresponding image collection.

***Processing of Circulating Tumour Cells (CTCs)***

Collected blood samples were microfuged at 0.4 G at 4⁰C for 5 minutes. The serum was discarded and 1 ml of Red Blood Cell Lysis buffer (8.2 g of Ammonium chloride, 1.0g of potassium hydrogen carbonate, and 0.38g of EDTA dissolved in 1 litre of filter sterilized Milli-Q™ water) was added. The sample was microfuged at 0.4 G at 4⁰C for 5 minutes. The aforementioned 2 steps were repeated 3 times. Subsequently the supernatant was discarded and the pellet was re-suspended with 1 ml of cold PBS before being microfuged at 0.4G at 4⁰C for 5 minutes. This step was repeated another 3 times. The final pellet was re-suspended in 500 µl of culture media (DMEM/F12 medium (1:1) supplemented with 5% horse serum and 4 mM glutamine) and pipetted into 6 cm culture dishes, and incubated at 37^o^C, 5% CO_2_ in a routine cell culture incubator for 7 days.

***Haematoxylin & Eosin staining and Imaging***

Numerical categories were assigned to the histological results to enable comparison among the 3 groups: 0= absence of DCIS and cancer, just normal breast tissue or inflammation (in DCIS cell-only group, see Figure 2 A &B); 1= presence of high-grade DCIS only (Figure 2 C); 2= presence of invasive carcinoma (grade 3) (Figure 2 D) + high-grade DCIS (with estimated invasive cancer <50% of entire tissue section); 3= presence of invasive carcinoma (grade 3) + high-grade DCIS (with estimated invasive cancer ≥50% of entire tissue section).

***Immunohistochemical staining***

Ku70 staining was performed on a Ventana Discovery XT autostainer (Ventana Medical Systems Inc, Roche Group) using anti-human Ku70 mouse monoclonal antibody (Abcam ab58150; 83 ng/ml). Sections were incubated with Cell Conditioning 1 solution (Ventana) for antigen retrieval followed by a primary antibody incubation of 32 minutes. The Ventana biotin-free, anti-mouse IgG detection system was used to localize the antibody (anti-Mouse HQ 16 min, anti-HQ HRP 16 minutes, DAB) and nuclei counterstained using Haematoxylin II (12 min) and Bluing regent (4 minutes) prior to cover slipping using DePeX. Cytokeratin 5 (CK-5) staining was performed manually. Sections were subjected to microwave antigen retrieval with pH 8.0 EDTA. Non-specific peroxidases were blocked with hydrogen peroxide. Prior to primary antibody (1:50; Leica, NCL-CK5, clone XM26) incubation for 1 hour at room temperature, non-specific proteins were blocked with CAS block (Thermo Fisher Scientific). Following biotinylated secondary antibody (1:150) incubation, signal was amplified using the VECTASTAIN Elite ABC Kit (Vector Laboratories). Specific staining was visualized with DAB and sections were counterstained with haematoxylin prior to cover slipping using DePeX.
