# Supplementary figures and images for: Mammographically dense human breast tissue stimulates MCF10DCIS.com progression to invasive lesions and metastasis
Source: Breast Cancer Res. 2016 Oct 25;18:106. doi: 10.1186/s13058-016-0767-4 (PMC5078949; doi:10.1186/s13058-016-0767-4)

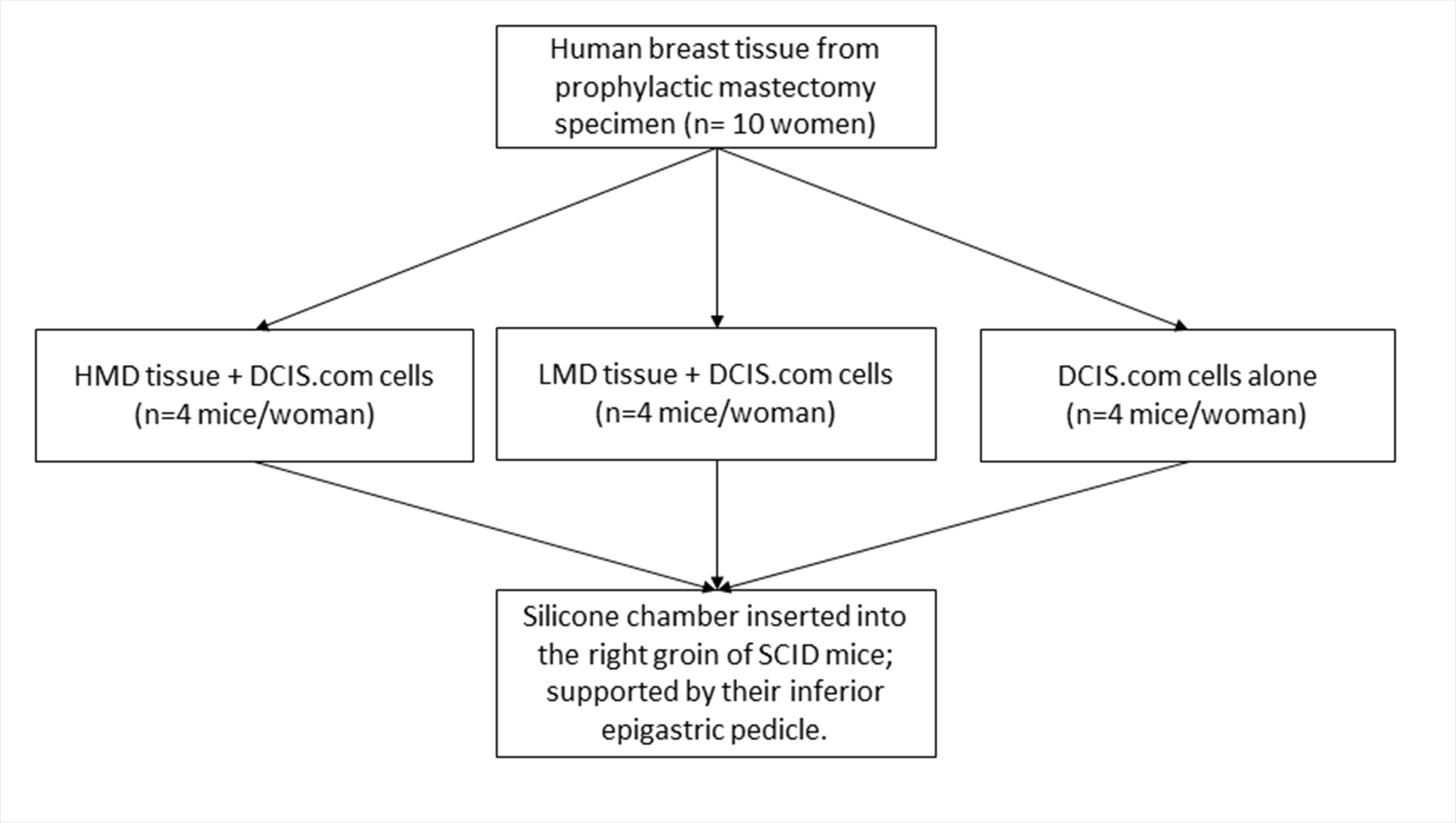

Supplement: Additional file 2: Figure S1. — The murine xenograft model. Schematic diagrams illustrate the use of 12 SCID mice associated with each patient’s tissue accrual and the allocation of 4 mice into DCIS.com + HMD, DCIS.com + LMD and DCIS.com-only groups. The schematic mouse shows a silicone chamber inserted in the groin with chamber material (in grey) vascularised by the inferior epigastric pedicle (in red). HMD High mammographic density, LMD low mammographic density, DCIS.com MFC10DCIS.com cells. (TIF 537 kb) [file 13058_2016_767_MOESM2_ESM.tif]

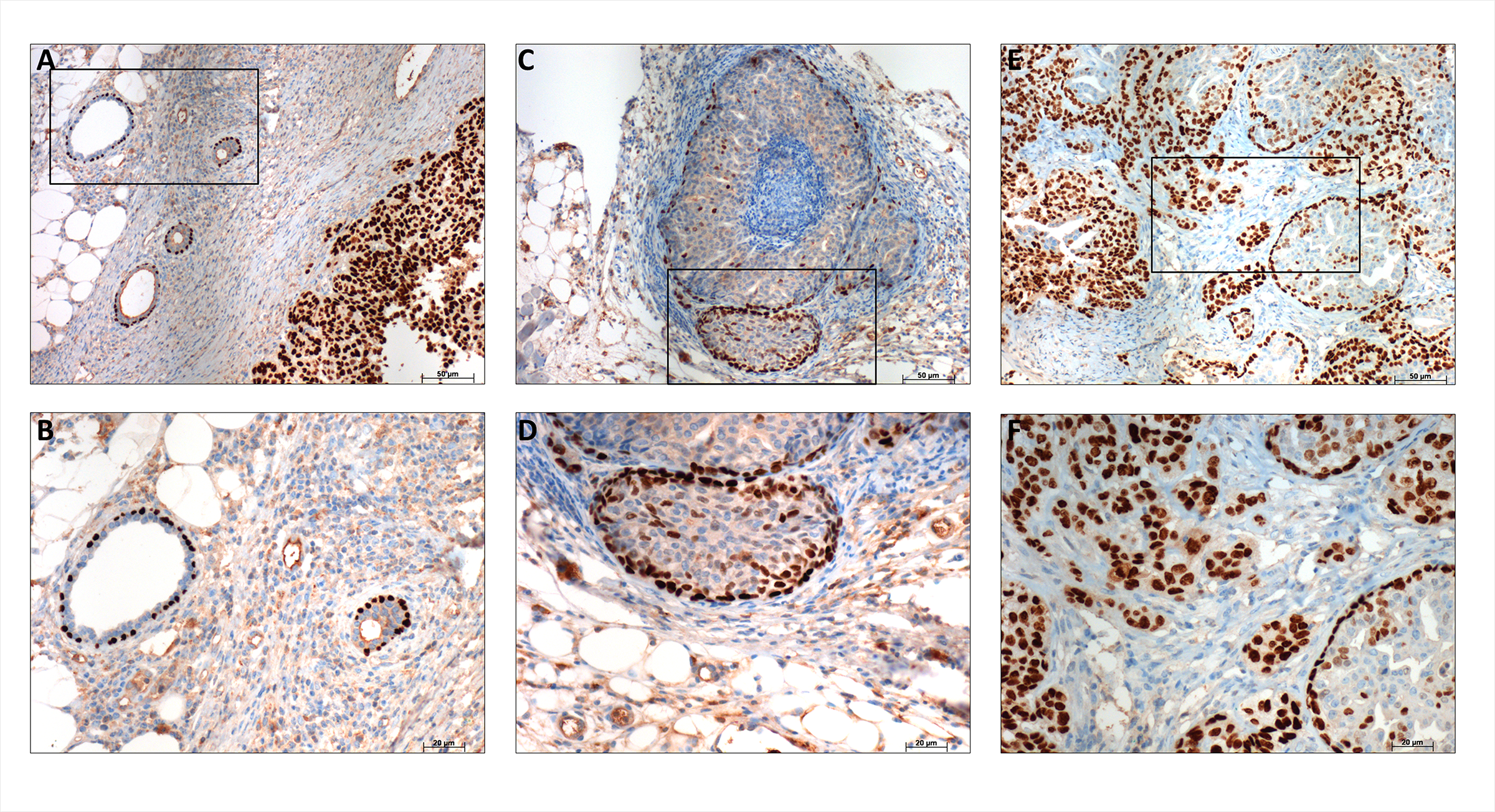

Supplement: Additional file 3: Figure S2. — p63 immunohistochemical nuclear staining. a Representative photomicrograph of punctate brown nuclear staining with p63 of normal human mammary glands adjacent to invasive tumour cells from a chamber explant at × 10 original magnification. b Normal mammary ducts shown in (a) at × 20 original magnification. c Representative photomicrograph of punctate brown nuclear staining with p63 of a ductal carcinoma in situ lesion from a chamber explant at × 10 original magnification. d Ductal carcinoma in situ lesion shown in (c) at × 20 original magnification. e Representative photomicrograph of punctate brown nuclear staining with p63 of invasive ductal carcinoma adjacent to ductal carcinoma in situ lesions at × 10 original magnification. f Invasive ductal carcinoma cells adjacent to ductal carcinoma in situ lesions shown in (e) at × 20 original magnification. (TIF 7517 kb) [file 13058_2016_767_MOESM3_ESM.tif]

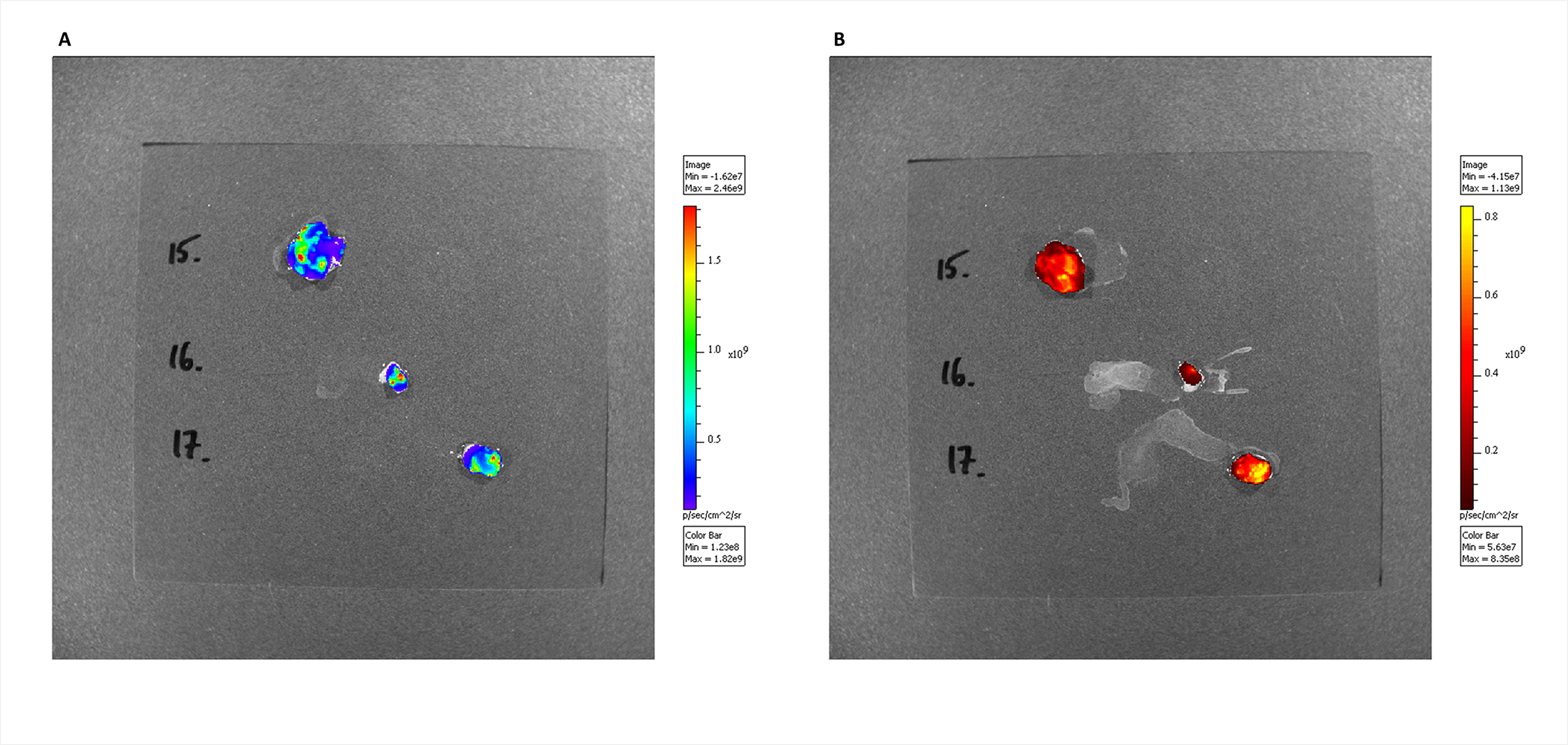

Supplement: Additional file 4: Figure S3. — Comparison of luciferase and mCherry imaging. a Representative luciferase imaging of three chamber explants. b Representative mCherry imaging of the same chamber explants shown in a. (TIF 2371 kb) [file 13058_2016_767_MOESM4_ESM.tif]
