# Supplementary material for: Mammographically dense human breast tissue stimulates MCF10DCIS.com progression to invasive lesions and metastasis
Source: Breast Cancer Res. 2016 Oct 25;18:106. doi: 10.1186/s13058-016-0767-4 (PMC5078949; doi:10.1186/s13058-016-0767-4)
Supplement: Additional file 5: Figure S4. — Correlation between participant demographics and DCIS.com cellular responses. The table outlines the individual demographic characteristics of each patient (n = 10). a Coloured bar graphs show patient-matched DCIS.com only (blue), DCIS.com + HMD (red) and DCIS.com + LMD (green) comparisons of chamber explant weights (a), chamber explant luciferase signals (b), mean histological category of chamber explants (c), mean number of CTCs/ml (d), mean number of metastasis-positive mouse organs (e) and mean luciferase signals for total metastases (f) for each patient. HMD high mammographic density, LMD low mammographic density, DCIS MCF10DCIS.com cells, CTC circulating tumour cells, BC breast cancer, + positive, N/A Not available. Triangles indicate data value of 0. BI-RADS score 1 = predominantly fat, 2 = scattered fibroglandular densities, 3 = heterogeneously dense, 4 = extremely dense. (PPTX 500 kb) [file 13058_2016_767_MOESM5_ESM.pptx]

## Slide 1
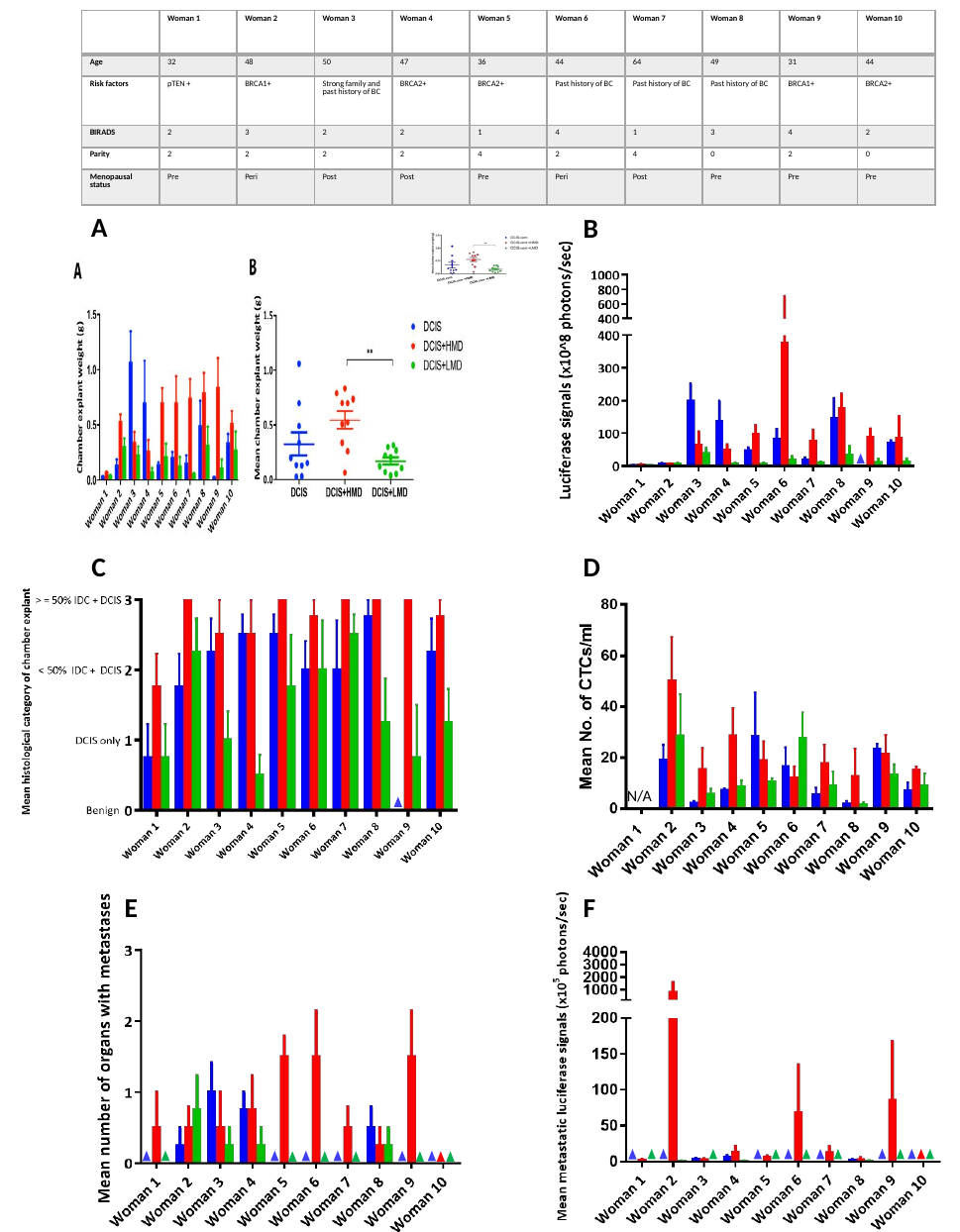

| | Woman 1 | Woman 2 | Woman 3 | Woman 4 | Woman 5 | Woman 6 | Woman 7 | Woman 8 | Woman 9 | Woman 10 |
| --- | --- | --- | --- | --- | --- | --- | --- | --- | --- | --- |
| Age | 32 | 48 | 50 | 47 | 36 | 44 | 64 | 49 | 31 | 44 |
| Risk factors | pTEN + | BRCA1+ | Strong family and past history of BC | BRCA2+ | BRCA2+ | Past history of BC | Past history of BC | Past history of BC | BRCA1+ | BRCA2+ |
| BIRADS | 2 | 3 | 2 | 2 | 1 | 4 | 1 | 3 | 4 | 2 |
| Parity | 2 | 2 | 2 | 2 | 4 | 2 | 4 | 0 | 2 | 0 |
| Menopausal status | Pre | Peri | Post | Post | Pre | Peri | Post | Pre | Pre | Pre |
A
B
C
D
N/A
E
F
